# Infinitesimal Dosing

**Published:** 1875-05

**Authors:** 


INFINTESIMAL DOSING.—Dr. John C. Peters, in his address on "Sects in Medicine," delivered before the New York Medico-Legal Society, says: "The Tartar physicians, or Llama doctors, have long superseded infintesimal doses, as, if they do not happen to have any medicine with them, they are by no means disconcerted, for they merely write the name of the remedy they wish to give on a little scrap of paper, moisten this with the saliva, roll it up into a pill, which the patient tosses down his throat with the same perfect confidence as he would aloes, assafœtida, or any other remedy. To swallow the name of the remedy, or to take the medicine itself, say the Tartar physicians, by any patient, comes to precisely the same thing. If paper is not at hand, the name of the drug is written with clay or chalk upon a board, which is then washed off, and the patient swallows the liquid."

Some of our physicians even dispense with this formality, and do not give any medicine at all. They certainly have the advantage over the Tartar doctors, as they save themselves some trouble and do not run the frightful risk of misspelling the name of the drug and perchance killing the patient.—*Medical Record.*

MALLEABLE glass that can be hammered is nothing new. Buggins has a glass still unbroken, though he has kept punch-in-it at intervals for several years.

THE New York *World* wants to know if a man with a cough is not a hackman.

